# Supplementary material for: Evaluation of surveillance-response interventions for Schistosoma haematobium elimination on Pemba Island, Tanzania: A 4-year intervention study with repeated cross-sectional surveys
Source: PLoS Negl Trop Dis. 2026 Feb 3;20(2):e0013947. doi: 10.1371/journal.pntd.0013947 (PMC12867264; doi:10.1371/journal.pntd.0013947)
Supplement: S1 Text — (PDF) [file pntd.0013947.s001.pdf]

Microhematuria prevalence in low-prevalence implementation units per year

In 2021, the baseline microhematuria prevalence in the 11 schools within the 15 low-prevalence areas was 3.0% (47/1554). Among all children, 0.4% (6/1554) had large microhematuria (S1A Figure). After the first period of surveillance-response interventions in these areas, the prevalence changed to 6.3% (105/1655) with 0.5% (9/1655) large microhematuria. In 2022, the microhematuria prevalence in the 14 schools of the 16 low-prevalence areas was 7.0% (147/2123). Among all children, 0.9% (19/2123) had large microhematuria. After the second period of surveillance-response interventions, the prevalence changed to 5.2% (117/2240) with 0.7% (16/2240) large microhematuria. In 2023, the microhematuria prevalence in the 14 schools located in the 17 low-prevalence areas was 5.0% (115/2287), and 0.4% (8/2287) of children had large microhematuria. After the third surveillance-response intervention period, the prevalence in these areas changed to 2.7% (73/2755) and 0.5% (13/2755) of children had large microhematuria.

In 2021, the baseline microhematuria prevalence in the 15 communities within the 15 low-prevalence areas was 5.5% (162/2969). Among all participants, 1.1% (31/2969) had large microhematuria (S1B Figure). After the first period of surveillance-response interventions in these areas, the prevalence changed to 13.3% (385/2928) and 1.1% (33/2928) of the tested population had large microhematuria. In 2022, the microhematuria prevalence in the 16 communities of the 16 low-prevalence areas was 11.2% (353/3175). Among the participants, 1.1% (34/3175) had large microhematuria. After the second period of surveillance-response interventions, the prevalence dropped to 10.0% (296/2981) and 1.0% (30/2981) of the participants had large microhematuria.

In 2023, the microhematuria prevalence in the 17 communities located in the 17 low-prevalence areas was 9.1% (297/3257), and 1.1% (35/3257) of the participants had large

microhematuria. After the third period of surveillance-response interventions, the prevalence in the 17 communities changed to 9.7% (300/3106) and 1.9% (58/3106) large microhematuria cases.
