# Supplementary material for: Evaluation of surveillance-response interventions for Schistosoma haematobium elimination on Pemba Island, Tanzania: A 4-year intervention study with repeated cross-sectional surveys
Source: PLoS Negl Trop Dis. 2026 Feb 3;20(2):e0013947. doi: 10.1371/journal.pntd.0013947 (PMC12867264; doi:10.1371/journal.pntd.0013947)
Supplement: S1 STROBE Checklist — (PDF) [file pntd.0013947.s003.pdf]

S1 STROBE Checklist

This filled checklist is based on the STROBE Statement-Checklist of items that should be included in reports of observational studies, developed by the STROBE Initiative. It is licensed under a Creative Commons Attribution 4.0 International License (CC BY 4.0).

	Item No.	Recommendation	Page No.	Relevant text from manuscript
**Title and abstract**	1	(*a*) Indicate the study's design with a commonly used term in the title or the abstract	1	Evaluation of surveillance-response interventions for *Schistosoma haematobium* elimination on Pemba Island, Tanzania: a 4-year intervention study with repeated cross-sectional surveys
		(*b*) Provide in the abstract an informative and balanced summary of what was done and what was found	3-4	Background: The Zanzibar islands, Tanzania, have eliminated schistosomiasis as a public health problem since 2017 and need to rethink their intervention strategies to ensure that the progress made is sustained and advanced. We evaluated the performance of a novel surveillance-response approach for interrupting *Schistosoma* transmission on Pemba, Tanzania, from 2020-2024.<br><br>Methodology: In low prevalence implementation units (IUs), surveillance-response interventions were implemented, which consisted of active and reactive case finding, treatment of *S. haematobium*-positive individuals, and reactive snail control. The performance of the surveillance-response interventions was measured by sensitivity, timeliness and impact on prevalence. Annual cross-sectional surveys were conducted in schools and households to estimate the total number of individuals infected with *S. haematobium* in the area and the proportion identified by the surveillance-response approach. Urogenital schistosomiasis was diagnosed by egg microscopy.<br><br>Principal Findings: Among the 20 IUs in the study area, 15, 16, and 17 were considered low-prevalence IUs in the intervention periods in 2021, 2022, and 2023, respectively. Across the intervention periods, 4.6% (707/15509) among the schoolchildren included in active surveillance were tested *S. haematobium*-positive and treated. During reactive surveillance, at water bodies 8.2% (10/122) and in households 9.9% (45/454) of individuals were found infected and treated. Moreover, 47 among the 262 waterbodies were treated with molluscicide. The overall sensitivity of the surveillance-response approach across the 3 periods was 43.1% (range: 14.4%-110.4%). The timeliness of reactive interventions was 2 weeks. In the low-prevalence IUs, the prevalence in schoolchildren changed from 0.5% (7/1552) in 2021 to 0.4% (6/1653) in 2022, from 0.6% (12/2123) in 2022 to 0.7% (15/2240) in 2023, and from 0.4% (8/2287) in 2023 to 1.0% (27/2755) in 2024 after surveillance-response implementation. The respective prevalence in community members was 0.5% (14/2969) in 2021 and 0.7% (19/2928) in 2022, 0.6% (18/3175) in 2022 and 0.3% (10/2979) in 2023, and 0.4% (12/3257) in 2023 and 0.7% (22/3106) in 2024.Conclusion: Surveillance-response interventions maintained the low *S. haematobium* prevalence, but interruption of transmission was not achieved. The overall sensitivity of the approach was moderate. Timeliness was very good but required strong communication and collaboration between the surveillance-response teams. To work on a larger scale, with good coverage and improved sensitivity, elimination programs will need a large number of well-trained staff and adequate tools for surveillance and response activities, data management, and communication.<br><br>Trial registration: ISRCTN, ISCRCTN91431493. Registered 11 February 2020, https://www.isrctn.com/ISRCTN91431493.

**Introduction**				
Background/rationale	2	Explain the scientific background and rationale for the investigation being reported	6-7	Schistosomiasis is a neglected tropical disease that affects more than 250 million people globally, particularly in sub-Saharan Africa [1]. The World Health Organization (WHO) has set the goal to eliminate schistosomiasis as a public health problem globally and eliminate infections in humans in specific countries by 2030 [2, 3]. Mass drug administration (MDA) with praziquantel has long been considered as the primary strategy for controlling and eliminating schistosomiasis [4]. Despite its success in reducing prevalence in many countries, MDA has limitations, including concerns about potential drug resistance, increasing treatment hesitancy in target populations due to the lack of prior testing and fear of side effects, and evolving challenges related to restricted drug availability and limited funding for the implementation of MDAs [3, 5-8]. Moreover, in areas that have successfully controlled schistosomiasis and achieved elimination as a public health problem (<1% of heavy intensity infections), only a few people are infected with *Schistosoma* parasites, and hence, only a small part of the population requires treatment [9-11].<br><br>In their new monitoring and evaluation framework for schistosomiasis published in 2024, the WHO flags that areas that have achieved elimination as a public health problem will enter a maintenance phase where a low prevalence of infection may still persist and where MDA at a reduced frequency may be necessary to maintain program gains and prevent recrudescence of infection [2, 12]. The WHO also indicates that surveillance will be essential until there is no more risk of a rebound of prevalence and intensity of infection [2, 12].<br><br>While details of a surveillance approach for schistosomiasis were not yet provided by the WHO in mid 2025, general guidelines for communicable disease surveillance-response systems were published two decades ago [12]. Surveillance-response is used as an intervention in other disease elimination programs, for example malaria [13]. Also for schistosomiasis, it has long been pointed out that to ensure that the progress made by control programs is sustained and advanced, countries and areas in the elimination phase must strengthen their health systems and participate in the development and use of integrated surveillance-response schemes [14]. It is suggested that surveillance, followed-up by public health actions consisting of response packages tailored to interruption of transmission in different settings, will help to effectively achieve the schistosomiasis control and elimination goals set by WHO for 2030 [3, 14, 15].<br><br>In many areas of the Zanzibar islands of the United Republic of Tanzania, urogenital schistosomiasis has been eliminated as a public health problem over the past decades [16-18]. The endemic landscape is nowadays very heterogeneous: most communities on the island have a very low or zero prevalence, but in some areas higher prevalences persist or rebound quickly after interventions [16]. Large-scale MDA across the islands seems no longer justified and new targeted intervention approaches are needed to ensure that the progress made is sustained and/or interruption of transmission accelerated.
Objectives	3	State specific objectives, including any prespecified hypotheses	7	The SchistoBreak project, implemented on Pemba Island from 2020 to 2024, tested a novel adaptive intervention approach that consisted of i) a comprehensive intervention package including MDA, snail control and behavior change communication in hotspot areas [19-21] and ii) novel surveillance-response interventions including active, passive and reactive case finding, treatment of *S. haematobium*-positive

				individuals, and reactive snail control in low-prevalence areas. Here, we evaluated the performance of the surveillance-response approach for its sensitivity, timeliness and impact on prevalence.
**Methods**				
Study design	4	Present key elements of study design early in the paper	9-10	The SchistoBreak study was designed as an intervention study with repeated cross-sectional surveys conducted in schools and households to monitor the prevalence of *S. haematobium* in the IUs and to assess the impact of interventions [19]. The fieldwork for the study was implemented from February 2020 until March 2024. Annual cross-sectional parasitological surveys were conducted in schools and households of all 20 IUs of the SchistoBreak study from November to February/March each year. Based on the results of the cross-sectional surveys, IUs were classified as low-prevalence IUs when they had a *S. haematobium* prevalence of <3% in schoolchildren and <2% in community members in the surveys, and as "hotspot" IUs when they had a *S. haematobium* prevalence of ≥3% in schoolchildren and ≥2% in community members. Interventions were implemented between the annual surveys in all IUs from May to October each year. The interventions were targeted at the micro-epidemiology, revealed in the annual surveys. In low-prevalence IUs, surveillance-response interventions were implemented, as described below. The multi-disciplinary interventions implemented in hotspot IUs are not the focus of this manuscript and are described elsewhere in detail [19, 24]. To be eligible for participation in the cross-sectional surveys or intervention activities of this study, individuals had to meet the following criteria: i) living or attending school in one of the IUs, ii) being aged ≥4 years, and iii) submitting the required informed consent forms.
Setting	5	Describe the setting, locations, and relevant dates, including periods of recruitment, exposure, follow-up, and data collection	9	The SchistoBreak study was conducted on Pemba Island, which is part of the semi-autonomous Zanzibar archipelago of the United Republic of Tanzania. Pemba is situated approximately 30 km off the coast of mainland Tanzania and is divided into four districts, which are subdivided into 129 small administrative areas, called shehias [22]. In the SchistoBreak study, each shehia represented an IU. The study included 20 IUs in two districts in the north of Pemba, Micheweni and Wete. According to the 2022 Tanzanian census, the study area had a population of about 95,000 inhabitants and a population growth rate of 2.5 [22]. Of the 20 IUs, 18 IUs had at least one public primary school. Most of these primary schools also included a nursery school. Additionally, in each IU, there were several Islamic schools, called madrassas. Finally, a total of 22 health facilities were located in the study area, including 20 primary health care units (PHCUs) and two district hospitals [23].
Participants	6	(*a*) *Cohort study*—Give the eligibility criteria, and the sources and methods of selection of participants. Describe methods of follow-up<br>*Case-control study*—Give the eligibility criteria, and the sources and methods of case ascertainment and control	10<br><br>10 & 11	To be eligible for participation in the cross-sectional surveys or intervention activities of this study, individuals had to meet the following criteria: i) living or attending school in one of the IUs, ii) being aged ≥4 years, and iii) submitting the required informed consent forms.<br><br>To start with active surveillance, in each low-prevalence IU, one primary school and one madrassa were selected. In each IU, the largest public primary school was chosen. The madrassa was selected based on risk and ensuring a wide coverage of the study area, considering the following indicators: The madrassa had i) a minimum of 50 students, ii) was located max. 500 m distant from a water body, and iii) was the madrassa farthest away from the selected public primary school. Once the schools were visited, the procedure was explained to all children in grades 3-5 in the primary school and to all children in the madrassa. On the first day of the study team's visit, the children were registered and handed out information sheets and consent

		selection. Give the rationale for the choice of cases and controls<br>*Cross-sectional study*—Give the eligibility criteria, and the sources and methods of selection of participants	11	forms to be given to their parents. On the second day, once signed informed consent forms had been submitted, children's demographic data were recorded, and they were invited to provide a fresh urine sample.<br><br>Subsequently, for reactive surveillance, the study team accompanied children who had a positive test result to their homes and to the freshwater bodies they had used in the past. At the child's household and the water bodies, all present individuals were invited to participate in the study and hence to answer a short questionnaire about demographics and to provide a fresh urine sample.
		(*b*) *Cohort study*—For matched studies, give matching criteria and number of exposed and unexposed<br>*Case-control study*—For matched studies, give matching criteria and the number of controls per case		NA
Variables	7	Clearly define all outcomes, exposures, predictors, potential confounders, and effect modifiers. Give diagnostic criteria, if applicable	14	During the cross-sectional surveys and surveillance interventions, urine samples were collected and transferred to the PHL-IdC on the day of collection. At the PHL-IdC, all urine samples were examined for microhematuria using Hemastix reagent strips, unless they had already been tested at the point of care during the intervention period. The intensity of microhematuria was graded according to the manufacturer's color chart into negative, trace, small (+), moderate (++), or large (+++). Additionally, urine samples were subjected to the urine filtration method. For this purpose, 10 ml of urine were passed through a 13 mm fabric filter (Sefar Ltd., Bury, United Kingdom), which was placed in a Swinnex plastic filter holder (Millipore, Merck KGaA, Darmstadt, Germany), using a plastic syringe. Subsequently, the filters were examined for the presence and quantity of *S. haematobium* eggs using a light microscope. Participants with 1 to 49 eggs per 10 ml of urine were considered to have a light-intensity infection, while individuals with 50 or more eggs per 10 ml of urine were considered to harbor a heavy-intensity infection [27].
Data sources/ measurement	8*	For each variable of interest, give sources of data and details of methods of assessment (measurement). Describe comparability of assessment		

		methods if there is more than one group
Bias	9	Describe any efforts to address potential sources of bias
Study size	10	Explain how the study size was arrived at

Continued on next page

Quantitative variables	11	Explain how quantitative variables were handled in the analyses. If applicable, describe which groupings were chosen and why	16-17	To evaluate the surveillance-response approach, its sensitivity was calculated per age group (children versus adults), per year, and overall. The sensitivity of surveillance interventions for infectious diseases is defined as "the proportion of cases of a disease or health condition detected by the surveillance system" [12, 28]. The sensitivity of the surveillance-response approach was determined with the following formula: $$\boldsymbol{Sensitivity} = \frac{N_{inf\ detected\ by\ surveillance}}{N_{inf}}$$ The total number of individuals infected with *S. haematobium* in the study area (N_{inf}) was unknown and was estimated with the following formula: $$N_{inf,i} = \left(\frac{P_{i,children} + P_{i+1,children}}{2} \times N_{children}\right) + \left(\frac{P_{i,adults} + P_{i+1,adults}}{2} \times N_{adults}\right)$$ Whereby the *S. haematobium* prevalence (P) is based on the results of repeated cross-sectional parasitological school and community surveys, and i represents the year of intervention. As the surveillance-response interventions were conducted midway between two annual cross-sectional surveys, the average prevalence of the survey before and the survey after the intervention phase was used as the reference prevalence, respectively ($N_{inf,2021}$ = estimated infections in 2021, $N_{inf,2022}$ = estimated infections in 2022, and $N_{inf,2023}$ = estimated infections in 2023). The population size (N) of the study area was determined based on data obtained from the 2022 Tanzania population census [22]. Based on the population growth rate of 2.5, the population sizes for the years 2021, 2023, and 2024 were estimated. To determine the number of school-aged children and adults in the population, percentages from the 2012 Tanzania population census were used [29], since the socio-demographic detailed numbers from the 2022 census were not yet publicly available. It was estimated that 40.7% of the population in the study area was aged 4-17 years, 45.3% were aged ≥18 years, and 14.0% were aged <4 years. The latter population group was not considered in the analysis for the sensitivity, since this age group was not included in the study.
Statistical methods	12	(*a*) Describe all statistical methods, including those used to control for confounding	16-18	To demonstrate the impact of the surveillance-response interventions, the prevalence of *S. haematobium* infections and microhematuria in the low-prevalence IUs was calculated per year, based on the annual cross-sectional school and community surveys.<br>To evaluate the surveillance-response approach, its sensitivity was calculated per age group (children versus adults), per year, and overall. The sensitivity of surveillance interventions for infectious diseases is defined as "the proportion of cases of a disease or health condition detected by the surveillance system" [12, 28]. The sensitivity of the surveillance-response approach was determined with the following formula: $$\boldsymbol{Sensitivity} = \frac{N_{inf\ detected\ by\ surveillance}}{N_{inf}}$$ The total number of individuals infected with *S. haematobium* in the study area (N_{inf}) was unknown and was estimated with the following formula: $$N_{inf,i} = \left(\frac{P_{i,children} + P_{i+1,children}}{2} \times N_{children}\right) + \left(\frac{P_{i,adults} + P_{i+1,adults}}{2} \times N_{adults}\right)$$

		Whereby the *S. haematobium* prevalence (P) is based on the results of repeated cross-sectional parasitological school and community surveys, and i represents the year of intervention. As the surveillance-response interventions were conducted midway between two annual cross-sectional surveys, the average prevalence of the survey before and the survey after the intervention phase was used as the reference prevalence, respectively ($N_{inf,2021}$ = estimated infections in 2021, $N_{inf,2022}$ = estimated infections in 2022, and $N_{inf,2023}$ = estimated infections in 2023). The population size (N) of the study area was determined based on data obtained from the 2022 Tanzania population census [22]. Based on the population growth rate of 2.5, the population sizes for the years 2021, 2023, and 2024 were estimated. To determine the number of school-aged children and adults in the population, percentages from the 2012 Tanzania population census were used [29], since the socio-demographic detailed numbers from the 2022 census were not yet publicly available. It was estimated that 40.7% of the population in the study area was aged 4-17 years, 45.3% were aged ≥18 years, and 14.0% were aged <4 years. The latter population group was not considered in the analysis for the sensitivity, since this age group was not included in the study.<br>For the calculation of P_{school} in 2021, multiple imputation was performed for missing urine filtration data using the mi package in R, and prevalence estimates were pooled. The imputation model included microhematuria results, results from the portable PCR machine, age, sex, and the location of data collection (school, household, or water body).<br>For the total count of individuals tested during reactive surveillance in households and at water bodies, only individuals who were identified based on positive *S. haematobium* or microhematuria results of index children were included in the analyses. Individuals in households and at water bodies who were identified solely based on a child's positive PCR test result in 2021 were not included in the analysis.<br>The timeliness of the surveillance-response approach was evaluated, defined as the reflection of "speed or delay between steps in a surveillance system" [28]. For the analysis, the time between the registration of children and the following steps was assessed: i) microhematuria testing, ii) urine filtration testing, iii) treatment, iv) registration of household members, v) registration of individuals at water bodies, and vi) reactive snail control. For assessment of timeliness, negative time values were observed between two steps, such as when a water body had already been followed up and/or surveyed for snails based on one child's information, and was then indicated by another child as well. In such cases, the negative time values were treated as zero.
	(*b*) Describe any methods used to examine subgroups and interactions	16	To demonstrate the impact of the surveillance-response interventions, the prevalence of *S. haematobium* infections and microhematuria in the low-prevalence IUs was calculated per year, based on the annual cross-sectional school and community surveys.<br>To evaluate the surveillance-response approach, its sensitivity was calculated per age group (children versus adults), per year, and overall.
	(*c*) Explain how missing data were addressed	17	For the calculation of P_{school} in 2021, multiple imputation was performed for missing urine filtration data using the mi package in R, and prevalence estimates were pooled. The imputation model included microhematuria results, results from the portable PCR machine, age, sex, and the location of data collection (school, household, or water body).

		(*d*) *Cohort study*—If applicable, explain how loss to follow-up was addressed<br>*Case-control study*—If applicable, explain how matching of cases and controls was addressed<br>*Cross-sectional study*—If applicable, describe analytical methods taking account of sampling strategy		NA
		(*e*) Describe any sensitivity analyses		
**Results**				
Participants	13*	(a) Report numbers of individuals at each stage of study—eg numbers potentially eligible, examined for eligibility, confirmed eligible, included in the study, completing follow-up, and analysed	18-19	Based on the annual cross-sectional school and community surveys conducted in the 20 IUs of the SchistoBreak study in 2021, 2022, and 2023, 15 IUs were considered low-prevalence IUs in the intervention periods in 2021, 16 in 2022, and 17 in 2023, respectively.<br>At baseline in 2021, 11 of the 15 low-prevalence IUs had a public primary school, where active surveillance was implemented (Figure 1). Furthermore, active surveillance was conducted in 15 madrassas. In 2022, 14 of the 16 low-prevalence IUs had public primary schools, where active surveillance started, in addition to 16 madrassas. In 2023, 15 of the 17 low-prevalence IUs had a public primary school, where active surveillance was implemented. However, one of these 15 schools had only recently opened, and only its nursery school and grades 1 and 2 were running. Since active surveillance was restricted to grades 3-5, only 14 public primary schools were part of the interventions. In the same year, active surveillance was also conducted in 17 madrassas.<br>For active surveillance, in 2021, a total of 3703 children were tested for *S. haematobium* and/or microhematuria in primary schools, of whom 214 (5.8%) were found positive. In madrassas, 56/592 (9.5%) children had a positive test result. In 2022, a total of 128/4455 children from primary schools and 25/817 (3.1%) children from madrassas were tested for *S. haematobium* and/or microhematuria in primary schools and identified as positive. In 2023, a total of 245/4928 (5.0%) children from primary schools and 39/1014 (3.8%) from madrassas had a positive test result. Across the intervention periods, a total of 707 children were tested positive for *S. haematobium* and/or microhematuria who were subsequently tracked to their households and to the water bodies they frequented as part of reactive surveillance (Figure 1). Table 1 shows the socio-demographic characteristics of the participants in active and reactive surveillance in low-prevalence IUs in the three study periods.
		(b) Give reasons for non-participation at each stage	19	Figure 1

		(c) Consider use of a flow diagram	19	Figure 1
Descriptive data	14*	(a) Give characteristics of study participants (eg demographic, clinical, social) and information on exposures and potential confounders	20	Table 1
		(b) Indicate number of participants with missing data for each variable of interest	19 & 21	Figure 1 &<br>In 2021, tracking children who were infected with *S. haematobium* and/or had microhematuria in urine through active surveillance to water bodies they had used, resulted in an additional 67 individuals who were followed-up at water bodies. Among them, urine filtration results were available for 66, of whom 5 (7.6%) tested positive for *S. haematobium*. In addition, 243 individuals were followed up in households. Of these, 210 individuals had urine filtration results, with 28 (13.3%) testing positive for *S. haematobium*. In 2022, 22 individuals were followed up at water bodies, with urine filtration results available for 21 of them. Among these, 2 (9.5%) tested positive for *S. haematobium*. Furthermore, 104 individuals were followed up in households. Of these, urine filtration results were available for 93 individuals, of whom 5 (5.4%) were tested positive for *S. haematobium*. In 2023, based on the tracked index cases from schools, 47 individuals were followed up at water bodies. For 35 of these, urine filtration results were available, of which 3 (8.6%) tested positive. Furthermore, 152 individuals were followed up at households. Among these, urine filtration results were available for 151, of whom 12 (7.9%) individuals in households were infected with *S. haematobium*. Across all years, 136 individuals were followed up at water bodies with 122 urine filtration results available, of which 10 (8.2%) were tested positive (Figure 2A). Furthermore, 499 individuals were followed-up in households with 454 urine filtration results, of whom 45 (9.9%) individuals were infected with *S. haematobium* (Figure 2B).
		(c) *Cohort study*—Summarise follow-up time (eg, average and total amount)	26-27	As shown in Figure 4, in the low-prevalence IUs, the median number of days between the registration of children in primary schools or madrassas and the examination of their urine samples for microhematuria and *S. haematobium* infection, respectively, was one day (range: 0-12 days and 0-18 days, respectively). The median number of days between the registration in school and the praziquantel treatment of positive children was seven days (range: 1-182). However, 90.2% of all positive children received praziquantel treatment within two weeks after their initial registration. The median time between the school registration and the household follow-up and water body follow-up was seven days (range: 1-182 days and 0-174 days, respectively). In 89.7% and 86.1%, the household follow-up and water body follow-up, respectively, was conducted within 14 days after the school registration of the children. The median time difference between initial school registration and snail surveys conducted at the water bodies indicated by the positive-tested children was 16 days (range: 0-44 days). In total, 30.0% of the water bodies were surveyed within two weeks after the initial school registration of the children in primary schools or madrassas.
Outcome data	15*	*Cohort study*—Report numbers of outcome events or summary measures over time		

		Case-control study—Report numbers in each exposure category, or summary measures of exposure		
		Cross-sectional study—Report numbers of outcome events or summary measures	*28-29*	In the baseline cross-sectional survey conducted in 2021, the *S. haematobium* prevalence in the 11 schools within the 15 low-prevalence areas was 0.5% (7/1552). Among all children, 0.1% (2/1552) had a heavy-intensity infection (Fig 5A). Microhematuria was found in 3.0% (47/1554) of the children (S1 Text and S1A Fig). After the first period of surveillance-response interventions in these areas, the prevalence dropped to 0.4% (6/1653) with 0.0% heavy-intensity infections. In 2022, the *S. haematobium* prevalence in the 14 schools of the 16 low-prevalence areas was 0.6% (12/2123). Among all children, 0.1% (2/2123) had a heavy-intensity infection. After the second period of surveillance-response interventions, the prevalence changed to 0.7% (15/2240) with 0.2% (4/2240) heavy-intensity infections. In 2023, the *S. haematobium* prevalence in the 14 schools located in the 17 low-prevalence areas was 0.4% (8/2287) and 0.1% (2/2287) of children had a heavy-intensity infection. After the third surveillance-response intervention period, the prevalence in these areas changed to 1.0% (27/2755) and 0.1% (3/2755) of children had a heavy-intensity infection in 2024.<br>In the baseline survey conducted in 2021, the *S. haematobium* prevalence in the 15 communities within the 15 low-prevalence areas was 0.5% (14/2969). Among all participants, 0.03% (1/2969) had a heavy-intensity infection (Fig 5B). Microhematuria was detected in 5.5% (162/2969) of the participants (S1 Text and S1B Fig). After the first period of surveillance-response interventions in these areas, the prevalence changed to 0.7% (19/2928) and 0.1% (2/2928) of the tested population had a heavy-intensity infection. In 2022, the *S. haematobium* prevalence in the 16 communities of the 16 low-prevalence areas was 0.6% (18/3175). Among the participants, 0.2% (5/3175) had a heavy-intensity infection. After the second period of surveillance-response interventions, the prevalence dropped to 0.3% (10/2979) and 0.03% (1/2979) of the participants had heavy-intensity infections. In 2023, the *S. haematobium* prevalence in the 17 communities located in the 17 low-prevalence areas was 0.4% (12/3257), and 0.03% (1/3257) of the participants had a heavy-intensity infection. After the third period of surveillance-response interventions, the prevalence in the 17 communities changed to 0.7% (22/3106) and 0.1% (3/3106) heavy-intensity infections in 2024.
Main results	16	(*a*) Give unadjusted estimates and, if applicable, confounder-adjusted estimates and their precision (eg, 95% confidence interval). Make clear which confounders were adjusted for and why they were included	24	Based on the prevalence of the cross-sectional school and community surveys conducted in 2021 and in 2022, and population size estimations from the national census, a total of 241 individuals were estimated to be infected with *S. haematobium* in the 15 low-prevalence IUs in 2021 (Fig 3). During the first surveillance-response intervention period in 2021, 150 individuals were tested positive for *S. haematobium* in the 15 low-prevalence IUs. However, our data imputation indicated that a total of 270 individuals would have been tested positive if we had tested all samples for *S. haematobium* using urine filtration. Based on the imputed number, the sensitivity of the surveillance interventions in 2021 was 114.2% (Table 3). In 2022, a total of 327 individuals were estimated to be infected with *S. haematobium* in the 16 low-prevalence IUs, based on the prevalence data retrieved from the school and community surveys conducted in 2022 and 2023. During the second intervention period in 2022, 48 individuals were tested *S. haematobium*-positive in the 16 low-prevalence IUs. Hence, the sensitivity of the surveillance-response interventions was 14.7%. In 2023, 443 individuals were estimated to be infected with *S. haematobium* in the 17 low-prevalence IUs. During the

		intervention period, 129 individuals were tested *S. haematobium*-positive, resulting in a sensitivity of the surveillance interventions of 29.1%. Across the three intervention periods, the overall sensitivity of the surveillance interventions was 44.8% (Table 3).
	(*b*) Report category boundaries when continuous variables were categorized	NA
	(*c*) If relevant, consider translating estimates of relative risk into absolute risk for a meaningful time period	NA

Continued on next page

Other analyses	17	Report other analyses done—eg analyses of subgroups and interactions, and sensitivity analyses	28	In 2021, 19 PHCUs tested 159 patients who reported symptoms of urogenital schistosomiasis for microhematuria as a proxy for *S. haematobium*. Among them, a total of 53 (33.3%) patients were microhematuria-positive. In 2022, 21 PHCUs participated in the SchistoBreak study and 130 (16.7%) among 778 individuals with symptoms of urogenital schistosomiasis had microhematuria. In 2023, there were 23 collaborating PHCUs who tested 780 patients for microhematuria, among whose 211 (27.1%) were positive.
**Discussion**				
Key results	18	Summarise key results with reference to study objectives	31	Our surveillance-response approach identified a considerable number of *S. haematobium*-infected individuals and water bodies with *Bulinus* that were subsequently treated with praziquantel and niclosamide, respectively. However, the sensitivity, i.e., the proportion of schistosomiasis cases detected by the surveillance system, varied substantially by intervention period. In 2021, the estimated sensitivity was very high (above 100%), and we observed more *S. haematobium* cases than we had estimated from the census data and cross-sectional survey results. In 2022 and 2023, the sensitivity was considerably lower (below 30%), and far fewer *S. haematobium*-infected individuals, in particular adults, were detected by active and reactive surveillance interventions than we should have identified according to the census data.
Limitations	19	Discuss limitations of the study, taking into account sources of potential bias or imprecision. Discuss both direction and magnitude of any potential bias	31-32	While many additional positive cases were identified through this test-treat-track-test-treat (5T) approach, it may have missed several positive community members who were not related to the index cases [30]. Second, the cases were identified by a single hematuria assessment and/or single urine filtration. Since the sensitivity of these diagnostic approaches is low, particularly in elimination settings, some infections may not have been detected [31, 32]. Third, the observed variability across the years and the overall only moderate sensitivity may be due to an overestimation of the number of positive individuals, as it was based on census-projected population data rather than survey-informed data. Fourth, infected individuals may have been missed because our visits in households and at the water bodies did not coincide with the times people were present at home or used the water bodies. Finally, individuals with a positive test result in our annual cross-sectional surveys were treated with praziquantel at the end of the survey for ethical reasons, which might have resulted in a lower actual number of cases during the intervention periods.
Interpretation	20	Give a cautious overall interpretation of results considering objectives, limitations, multiplicity of analyses, results from similar studies, and other relevant evidence	32-33	Hence, while the surveillance-response approach allowed an effective identification and subsequent treatment of positive-tested individuals, its ability to detect all cases in the study area remained limited. The sensitivity of the approach likely could have been increased with a higher coverage, e.g., by screening more children in more schools in the study area. Our findings are similar to those of malaria research, where a high surveillance coverage was identified as one of the key gaps to be filled on the way to elimination [33, 34]. A malaria study conducted in Zanzibar implemented a malaria case notification platform collecting detailed data on all confirmed malaria cases from public and private health facilities, allowing for a prompt testing of household members of positive-tested individuals [35]. For schistosomiasis, no comparative system is in place in Zanzibar, but since urogenital schistosomiasis results in less intense symptoms and morbidity than malaria, and people do not necessarily consult health facilities for treatment, additional active case identification and notification systems are needed to ensure a rapid response and increase the sensitivity of surveillance-response activities.<br>Indeed, across the 4 study years, the 23 health facilities involved in the SchistoBreak project for passive surveillance, identified 1738 individuals with symptoms that may be caused by *S. haematobium* infections

				per study year. Among those, 394 (23.0%) had microhematuria and were hence treated with praziquantel. While these numbers very likely contributed to maintaining the low prevalence and health facilities should be part of an effective schistosomiasis surveillance-response system, they cannot be the only main component for case identification and notification. Of note, for health facilities to play an important role in passive surveillance for schistosomiasis elimination, their capacities need to be improved by regular staff training, and an unbroken supply of (yet unavailable) accurate point-of-care diagnostics and praziquantel for the treatment of cases [36].
Generalisability	21	Discuss the generalisability (external validity) of the study results	33	As for malaria and similar to our 5T approach, schistosomiasis cases identified in health facilities could also be used as index cases to identify additional cases, as done in a test-and-treat study conducted in Egypt [37].
**Other information**				
Funding	22	Give the source of funding and the role of the funders for the present study and, if applicable, for the original study on which the present article is based	37	Funding for the study has been obtained from the Swiss National Science Foundation (SNSF; Bern, Switzerland) via a PRIMA grant (PR00P3_179753) of SK. The funders had no role in study design, data collection and analysis, decision to publish, or preparation of the manuscript.

*Give information separately for cases and controls in case-control studies and, if applicable, for exposed and unexposed groups in cohort and cross-sectional studies.

Note: An Explanation and Elaboration article discusses each checklist item and gives methodological background and published examples of transparent reporting. The STROBE checklist is best used in conjunction with this article (freely available on the Web sites of PLoS Medicine at http://www.plosmedicine.org/, Annals of Internal Medicine at http://www.annals.org/, and Epidemiology at http://www.epidem.com/). Information on the STROBE Initiative is available at www.strobe-statement.org.
