# Supplementary material for: Evaluation of surveillance-response interventions for Schistosoma haematobium elimination on Pemba Island, Tanzania: A 4-year intervention study with repeated cross-sectional surveys
Source: PLoS Negl Trop Dis. 2026 Feb 3;20(2):e0013947. doi: 10.1371/journal.pntd.0013947 (PMC12867264; doi:10.1371/journal.pntd.0013947)
Supplement: Attachment — Submitted filename: PNTD-D-25-00990_Response to Reviewers_FINAL_21.11.2025.pdf [file pntd.0013947.s007.pdf]

Trippler et al. **“**Evaluation of surveillance-response interventions for *Schistosoma haematobium* elimination on Pemba Island, Tanzania: a 4-year intervention study with repeated cross-sectional surveys” (Your reference no. PNTD-D-25-00990) **– Point-by-Point response**

Allschwil, 21 November 2025

Dear Dr Liang,

We were delighted to receive your e-mail dated 24 September 2025, which provided valuable feedback from two reviewers regarding the afore mentioned manuscript. In the meantime, we carefully revised the manuscript in light of the reviewers’ comments and suggestions and feel that the overall quality of our manuscript has been further improved.

Below, please find our point-by-point response, clearly indicating how and where in the manuscript changes have been made (line numbers referring to the tracked changed version of the manuscript). To assist you in readily tracking our amendments in the manuscript, we have marked all changes made by using track changes. Additionally, we submit a clean version of the revised manuscript.

We very much hope that this revised version is now suitable for publication in *PLoS Neglected Tropical Diseases*. Hence, we look forward to your further disposition.

Yours sincerely,

Lydia Trippler and Stefanie Knopp

(on behalf of all authors)

****

Dear Dr. Knopp,

Thank you for submitting your manuscript to PLOS Neglected Tropical Diseases. After careful consideration, we feel that it has merit but does not fully meet PLOS Neglected Tropical Diseases's publication criteria as it currently stands. Therefore, we invite you to submit a revised version of the manuscript that addresses the points raised during the review process.

We thank you and the two external reviewers very much indeed for the thorough appraisal of our work and the overall positive feedback. We tried our level best to respond to each point and amended the manuscript accordingly. Please find our point-by-point response below.

Please submit your revised manuscript within 60 days Nov 22 2025 11:59PM. If you will need more time than this to complete your revisions, please reply to this message or contact the journal office at plosntds@plos.org. When you're ready to submit your revision, log on to https://www.editorialmanager.com/pntd/ and select the 'Submissions Needing Revision' folder to locate your manuscript file.

We have submitted our revised manuscript along with a point-by-point response to the reviewers' comments on November 21, 2025.

Please include the following items when submitting your revised manuscript:

* A rebuttal letter that responds to each point raised by the editor and reviewer(s). You should upload this letter as a separate file labeled 'Response to Reviewers'. This file does not need to include responses to any formatting updates and technical items listed in the 'Journal Requirements' section below.

We have uploaded a "Response to Reviewers" rebuttal letter, which responds to each point raised by the reviewers to the *PloS Neglected Tropical Diseases* online system.

* A marked-up copy of your manuscript that highlights changes made to the original version. You should upload this as a separate file labeled 'Revised Manuscript with Track Changes'.

We have uploaded a marked-up copy of our manuscript that highlights changes made to the original version using track changes and labelled it "Revised Manuscript with Track Changes".

* An unmarked version of your revised paper without tracked changes. You should upload this as a separate file labeled 'Manuscript'.

We have uploaded an unmarked version of our revised paper without tracked changes and labelled it "Manuscript".

If you would like to make changes to your financial disclosure, competing interests statement, or data availability statement, please make these updates within the submission form at the time of resubmission. Guidelines for resubmitting your figure files are available below the reviewer comments at the end of this letter.

We did not change our financial disclosure, competing interests statement, or data availability statement.

We look forward to receiving your revised manuscript.

Kind regards,

Song Liang
Academic Editor
PLOS Neglected Tropical Diseases

Francesca Tamarozzi
Section Editor
PLOS Neglected Tropical Diseases

Shaden Kamhawi
co-Editor-in-Chief
PLOS Neglected Tropical Diseases
orcid.org/0000-0003-4304-636XX

Paul Brindley
co-Editor-in-Chief
PLOS Neglected Tropical Diseases
orcid.org/0000-0003-1765-0002

****

REVIEWER REPORTS

Reviewer #1: The most pressing issue is the anomalous sensitivity estimate of 114.2% in 2021, which substantially undermines the credibility of the primary outcome and overall study conclusions. The authors must provide a transparent, quantitative explanation for how sensitivity could exceed 100% or revise/remove this calculation. Without resolution, the validity of the results cannot be assured.

We thank Reviewer #1 for drawing attention to the anomalous sensitivity estimate of 114.2% in 2021. This value likely arose because during active surveillance conducted in schools in 2021, only part of the collected urine samples were tested with urine filtration microscopy (**revised manuscript, lines 226-230**). To calculate the sensitivity using urine filtration microscopy as reference test (see our response to the next comment), we had to impute date from the available Hemastix and PCR results in 2021 (**revised manuscript, lines 379-382**), which affected the estimated number of identified *S. haematobium*-infected individuals for the intervention period in 2021 and hence impacted on the estimated sensitivity. To address this concern, we added information to Table 3, presenting the overall sensitivity of the surveillance system based i) on the imputed and unimputed data from the intervention periods in 2021, 2022 and 2023 and ii) solely on non-imputed data from the intervention periods in 2022 and 2023,

from which complete urine filtration data are available **(revised manuscript, Table 3)**. Moreover, we amended the text of the Results section as follows:

"Across the two intervention periods (2022 and 2023), for which complete urine filtration data were available and no data were imputed, the overall sensitivity of the surveillance interventions was 23.0%, with estimates ranging from 20.9% to 57.5% across the combined sensitivity analyses scenarios (Table 3)." **(revised manuscript, lines 507-510)**

For completeness, we also kept the sensitivity estimates for 2021 and 2021-2023, respectively, in Table 3 (**revised manuscript, lines 519-524)**.

Also in the discussion, we indicate that the high estimate for 2021 is likely due to data imputation: "However, the sensitivity, i.e., the proportion of schistosomiasis cases detected by the surveillance system, varied substantially by intervention period. In 2021, likely caused by data imputation, the estimated sensitivity was very high (above 100%), and we observed more *S. haematobium* cases than we had estimated from the census data and cross-sectional survey results." (**revised manuscript, lines 601-605)**.

Closely related is the inconsistent use of diagnostic methods across study years. Although PCR was introduced in 2021, results are presented as aggregated "positive" rates without identifying a clear reference standard or accounting for differences in diagnostic yield. The authors need to define the diagnostic gold standard explicitly, explain how varying methods influenced sensitivity estimates, and ensure that comparisons across years are both transparent and scientifically defensible.

We thank Reviewer #1 for this valuable comment. The diagnostic methods applied in this study were used consistently in the four annual cross-sectional surveys, where we applied a single urine filtration microscopy for *S. haematobium* egg detection and Hemastix reagent strips for haematuria assessment for all sample examinations in schools and communities. Also, in the intervention periods in 2022 and 2023, urine filtration and Hemastix were used for active and reactive surveillance. In the first intervention period in 2021, however, Hemastix and PeakPCR were used for active surveillance in schools, while urine filtration microscopy was conducted only on a subsample of participants (**revised manuscript, lines 379-382)**. For reactive surveillance, both urine filtration microscopy and Hemastix were employed in all intervention periods.

The analysis for the sensitivity of the surveillance system is based on urine filtration results only. For clarification, we have added the following details to the manuscript: "To evaluate the surveillance-response approach, its sensitivity was calculated per age group (children versus adults), per year, and overall, using urine filtration microscopy as diagnostic reference standard. "(**revised manuscript, lines 345-347**).

Since in 2021, urine filtration microscopy was only conducted on part of the collected urine samples, we had to impute data from PCR and Hemastix results for this intervention period,

which likely resulted in an overestimate of the sensitivity of the surveillance system in this year (and hence also an overestimate of the overall sensitivity results for 2021-2023). To address this concern, we amended Table 3 and the text in the manuscript as described above, in our response to the first comment of Reviewer#1.

A further concern is the sensitivity calculation's reliance on an estimated infected population (N_inf) that the authors themselves note may be "overestimated." This weak foundation makes the sensitivity values questionable. The authors should provide stronger justification for how N_inf was determined, perform sensitivity analyses to show how different assumptions affect the results, and clearly acknowledge limitations if uncertainty remains. Together, these issues represent essential revisions needed to meet PLOS NTD's standards for methodological rigor, validity, and clarity.

We agree with the reviewer that, as stated, N_inf may be overestimated. We have now run a sensitivity analysis with different assumptions, as explained in the Methods as follows:

"To assess robustness of the sensitivity of the surveillance approach, we varied two key assumptions in a sensitivity analysis: i) estimated population sizes were scaled by factors of 0.8, 0.9, 1, 1.1 (representing -20%, -10%, 0%, 10% change relative to projected census estimates), and ii) mid-period prevalence was scaled by factors of 1, 0.75, 0.5 (representing reductions of 0%, 25%, 50% relative to the cross-sectional mean prevalence), reflecting uncertainty in population denominators and lower true prevalence at the time of surveillance due to post-survey treatment." (**revised manuscript, lines 371 -377**).

Furthermore, we have provided the ranges of sensitivity based on these assumptions in the Results section as follows:

"Based on the prevalence of the cross-sectional school and community surveys conducted in 2021 and 2022, and population size estimations from the national census, a total of 241 individuals were estimated to be infected with *S. haematobium* in the 15 low-prevalence IUs in 2021 (Fig 3). During the first surveillance-response intervention period in 2021, 150 individuals tested positive for *S. haematobium* based on urine filtration microscopy of a sub-group of individuals in the 15 low-prevalence IUs. However, the data imputation indicated that a total of 270 individuals would have been tested positive if we had tested all samples for *S. haematobium* using urine filtration microscopy. Based on the imputed number of 270 positive individuals, the sensitivity of the surveillance interventions in 2021 was 114.2%, and varied between 103.8% and 285.5% across sensitivity analyses scenarios (Table 3). In 2022, a total of 327 individuals were estimated to be infected with *S. haematobium* in the 16 low-prevalence IUs, based on the prevalence data retrieved from the school and community surveys conducted in 2022 and 2023. During the second intervention period in 2022, 48 individuals tested *S. haematobium*-positive based on urine filtration in the 16 low-prevalence IUs. Hence, the sensitivity of the surveillance-response interventions was 14.7%, and varied between 13.4% and 36.8% across sensitivity analyses scenarios. In 2023, 443 individuals were estimated to be

infected with *S. haematobium* in the 17 low-prevalence IUs. During the intervention period, 129 individuals tested *S. haematobium*-positive based on urine filtration, resulting in a sensitivity of the surveillance interventions of 29.1%, which varied between 26.4% and 72.8% across sensitivity scenarios. Across the three intervention periods (2021, 2022 and 2023), the overall sensitivity of the surveillance interventions was 44.8%, with sensitivity estimates ranging from 40.7% to 112% across the combined sensitivity analyses scenarios. Across the two intervention periods (2022 and 2023), for which complete urine filtration data were available, and no data were imputed, the overall sensitivity of the surveillance interventions was 23.0%, with sensitivity estimates ranging from 20.9% to 57.5% across the combined sensitivity analyses scenarios (Table 3)." (**revised manuscript, lines 485-510**).

Lastly, the manuscript also briefly mentions different treatment referral pathways (point-of-care versus referral), but their implications for case detection and intervention effectiveness are underdeveloped. Expanding this discussion would help clarify the programmatic impact of these approaches and enhance the manuscript's contribution to public health practice

During active surveillance in schools and passive surveillance in health facilities, individuals that were *S. haematobium* and/or microhaematuria-positive received treatment with praziquantel that was administered at the point-of-care and directly observed by the study team or health facility staff, respectively (**revised manuscript, lines 233-235 and 273-276**). During reactive surveillance in households and at waterbodies, participants were either treated directly at the point-of-care by the study team when microhaematuria-positive, or were treated during an arranged meeting with the study team or referred to a health facility for treatment, when the *S. haematobium* infection was only identified in the laboratory (**revised manuscript, lines 242-246**). In case patients were referred to a health centre, the compliance with treatment was not double-checked. The implications of potential incompliance with treatment on intervention effectiveness is now briefly highlighted in the discussion as follows: "Besides the low to moderate sensitivity of the surveillance-response system to detect and treat cases, also incompliance with praziquantel treatment of infected individuals may have impacted on intervention effectiveness. While infected individuals identified during active and passive surveillance received directly observed treatment at the point-of-care, several infected individuals identified by reactive surveillance were referred to health facilities for treatment. In case these individuals did not seek treatment, they may have contributed to maintaining transmission." (**revised manuscript, lines 693-699**).

Moreover, we added compliance in our conclusion: "Clearly, to achieve complete interruption of *Schistosoma* transmission, in addition to treatment of infected individuals and mollusciciding against intermediate host snails, measures that prevent reinfection such as improvement of the water, sanitation and hygiene (WASH) infrastructure and sustainable behavior change communication to increase compliance with interventions and treatment will be required [1-3]." (**revised manuscript, lines 706-711**).

Reviewer #2: The introduction is too brief and should be strengthened by including recent data on the burden of schistosomiasis in sub-Saharan Africa and its contribution to global prevalence, as well as a short history of control in Zanzibar leading up to elimination.

In line with the comments of Reviewer #2, we have expanded the introduction by including recent global burden of disease results and additional information about the history of schistosomiasis control in Zanzibar. With regard to the latter, we would also like to draw attention to a recent systematic review entitled "The long road to schistosomiasis elimination in Zanzibar: A systematic review covering 100 years of research, interventions and control milestones" published in 2023 [4]." published in 2023 (**revised manuscript, reference 18**).

Specifically, we have amended the introduction by the following information:

"The global burden due to schistosomiasis was estimated at 1.86 million disability adjusted life years (DALYs) in 2021 [5]. Most people affected by schistosomiasis live in Africa, where the burden was estimated at 1.57 million DALYs in 2021 [5]." (**revised manuscript, lines 89-92**).

"The Zanzibar Islands of the United Republic of Tanzania look back to a long history of research and interventions against urogenital schistosomiasis [4]. Over the past decades, the prevalence of and morbidity due to *S. haematobium* were substantially reduced [4]. Elimination efforts started in 2012, with the Zanzibar Elimination of Schistosomiasis Transmission (ZEST) project [6,7], where, in addition to island-wide MDA in schools and communities, also behavior change communication and snail control interventions were implemented [7,8]. In 2017, in many areas of the islands, urogenital schistosomiasis had been eliminated as a public health problem [4,8,9]. (**revised manuscript, lines 120-130**).

The reviewer also requests clarification on why only Pemba was selected for the study, while Unguja—the larger and more populated island—was not included. Relatedly, more explanation is needed on the criteria used to select schools: when the manuscript says the "largest schools" per implementation unit were chosen, does this refer to population size or physical area? Similarly, while the rationale for choosing madrassas is clear, that for schools is not.

The study was implemented in Pemba (and not in Unguja) first and foremost due to limited funding, which was not sufficient to include both islands, but only 20 administrative areas (shehias) in the North of Pemba Island. Second, the Swiss Tropical Public Health Institute has a long-standing and very fruitful research partnership with the Public Health Laboratory – Ivo de Carneri on Pemba, which was an ideal and well-equipped basis to conduct solid research.

We thank the reviewer for the suggestion to clearly define in the manuscript the indicators based on which the primary schools were chosen. We have now included a definition of "largest school" in the revised manuscript: "In each IU, the largest public primary school was chosen, defined as the school with the highest number of enrolled children among all public primary schools in the IU." (**revised manuscript, lines 211-213**).

Several methodological and programmatic concerns also need to be addressed. The reviewer highlights that patient compliance with praziquantel treatment is often a major barrier to control and asks whether additional initiatives were undertaken to ensure patients actually received treatment.

We thank the Reviewer#2 for this insightful comment and fully agree that participant compliance with treatment is crucial for achieving effective control and interruption of *Schistosoma* transmission.

In hotspot implementation units, which are not the focus of this manuscript but of an already published piece [2], at least annual MDA was conducted by the Zanzibar Neglected Tropical Diseases (NTD) programme. In schools, teachers and staff of the NTD team directly observed praziquantel treatment of students. Hence, we expect a very high coverage and compliance. In communities, a door-to-door approach was used, where drug distributors provided praziquantel for all eligible household members. Here, compliance is probably considerably lower than by the directly observed treatment approach used in schools, as revealed in earlier studies [10].

In the low-prevalence implementation units that were the focus of the surveillance-response approach described in the present manuscript, only *S. haematobium* and/or microhaematuria-positive individuals were treated with praziquantel. In the absence of a highly sensitive and specific point-of-care test for *S. haematobium* diagnosis, for test-and-treat in households and at waterbodies, we used Hemastix reagent strips to test for microhaematuria as a proxy for infection. Microhaematuria-positive individuals were offered praziquantel directly at the place of testing (**revised manuscript, lines 242-243**). Those who tested microhaematuria-negative but were found infected with *S. haematobium* through urine filtration microscopy in the laboratory were followed up for praziquantel treatment. In such cases, individuals were contacted and advised to meet with the study team to receive praziquantel. When this was not feasible, they were instructed to obtain the drugs at the nearest health facility supplied with praziquantel through the study team (**revised manuscript, lines 243-246**). As the health facilities participating in passive surveillance were part of the study activities, the study team was aware of where praziquantel was available. While we cannot guarantee that all individuals referred to the health facilities ultimately took the drugs, every effort was made to ensure access to treatment and to inform participants about their infection status. We have added the following parts to the discussion to indicate the effect that incompliance may have had on intervention effectiveness:

During active surveillance in schools and passive surveillance in health facilities, individuals that were *S. haematobium* and/or microhaematuria-positive received treatment with praziquantel that was administered at the point-of-care and directly observed by the study team or health facility staff, respectively (**revised manuscript, lines 233-235 and 273-276**). During reactive surveillance in households and at waterbodies, participants were either treated directly at the point-of-care by the study team when microhaematuria-positive, or were treated during an arranged meeting with the study team or referred to a health facility for treatment, when the *S. haematobium* infection was only identified in the laboratory (**revised manuscript, lines 242-246**). In case patients were referred to a health centre, the compliance with treatment was not double-checked. The implications of potential incompliance with treatment on intervention

effectiveness is now briefly highlighted in the discussion as follows: “Besides the low to moderate sensitivity of the surveillance-response system to detect and treat cases, also incompliance with praziquantel treatment of infected individuals may have impacted on intervention effectiveness. While infected individuals identified during active and passive surveillance received directly observed treatment at the point-of-care, several infected individuals identified by reactive surveillance were referred to health facilities for treatment. In case these individuals did not seek treatment, they may have contributed to maintaining transmission.” (**revised manuscript, lines 693-699**).

Moreover, we added compliance in our conclusion: “Clearly, to achieve complete interruption of *Schistosoma* transmission, in addition to treatment of infected individuals and molluscicidingagainst intermediate host snails, measures that prevent reinfection such as improvement of the water, sanitation and hygiene (WASH) infrastructure and sustainable behavior change communication to increase compliance with interventions and treatment will be required [1-3].” (**revised manuscript, lines 706-711**).

They also raise concerns about the use of chemical molluscicides. Specifically, the authors should explain why niclosamide was applied even in areas where no snails were detected, despite potential ecological risks, and whether any assessment was made of the snail control method’s impact on aquatic ecosystems or steps were taken to prevent human exposure when communities use these waters for domestic purposes. The manuscript also mentions that environmental data (water chemistry, vegetation type) were collected, but these variables are not discussed in relation to snail presence or abundance and should be incorporated.

We thank the reviewer for this valuable comment. The WHO recommends the use of the molluscicide niclosamide for schistosomiasis elimination and to apply it in known transmission sites [11]. The aim of our study was to break transmission of schistosomiasis. Hence, we applied niclosamide to known habitat areas of the intermediate host snail *Bulinus* at places where humans have water contact (**see revised manuscript, lines 254-256**). If no *Bulinus* were found at a present or past survey, niclosamide was not applied (**see revised manuscript, lines 261-262**). We agree that the use of chemical molluscicides may potentially have extended effects on non-targeted other species. In our study, however, we did not assess the direct impact of snail control on the aquatic ecosystem. The leaders of the communities where snail control was conducted were informed about the procedures of mollusciciding at an annual meeting at PHL-IdC and in addition by the research team who conducted the mollusciciding in the days before niclosamide application and asked to extent the information to their community.

Regarding the variables of water chemistry and vegetation type and their relation to snail presence and abundance, we kindly refer the reviewer to one of our published manuscripts entitled “Impact of chemical snail control on intermediate host snail populations for urogenital schistosomiasis elimination in Pemba, Tanzania: findings of a 3-year intervention study”, published in 2024 [12]”, (**revised manuscript, reference 23**). The results are not the focus of the present manuscript, but we refer to the separate publication: “Details of snail surveys and control are provided elsewhere [12].” (**revised manuscript, lines 262-263**).

Finally, the reviewer requests clarification on how the outcome of passive surveillance interventions was measured (e.g., whether diagnosed cases increased following the intervention). They also note missing prevalence data for primary school children in 2022 (line 389), which should be included. Addressing these points will strengthen the manuscript's methodological rigor and provide important context for interpreting the intervention results

We thank the reviewer for this important comment. The main objective of the study was to interrupt *S. haematobium* transmission through a combination of interventions, including passive surveillance in health facilities. Passive surveillance was therefore not evaluated as a stand-alone intervention, but rather implemented as an integral component of the overall approach to ensure treatment of as many infected individuals as possible.

We are very grateful to the reviewer for pointing out that we did not indicate the percentage of positive-tested children in the primary schools for 2022. We have now added the percentage and the respective sentence reads: "In 2022, a total of 128/4455 (2.9%) children from primary schools and 25/817 (3.1%) children from madrassas were tested for *S. haematobium* and/or microhematuria in primary schools and identified as positive." (**revised manuscript, lines 414-416**).

<u>Comments</u>

1. Line 87: Authors have pointed out that sub-saharan African is the highly affected region. In view of this, it will be worthy including recent data that portrayed the magnitude of the disease in the mentioned region and its contribution to the global schistosomiasis prevalence.

We thank the reviewer for the comment and have added the following information: "The global burden due to schistosomiasis was estimated at 1.86 million disability adjusted life years (DALYs) in 2021 [5]. Most people affected by schistosomiasis live in Africa, where the burden was estimated at 1.57 million DALYs in 2021 [5]." (**revised manuscript, lines 89-92**).

2. The introduction section is very brief; I therefore suggest the inclusion of a brief history of schistosomiasis control in Zanzibar which consequently led to the diseases elimination.

We agree with the reviewer that the introduction deserves more information about the history of schistosomiasis control in Zanzibar and added the following information: "The Zanzibar Islands of the United Republic of Tanzania look back to a long history of research and interventions against urogenital schistosomiasis [4]. Over the past decades, the prevalence of and morbidity due to *S. haematobium* were substantially reduced [4]. Elimination efforts started in 2012, with the Zanzibar Elimination of Schistosomiasis Transmission (ZEST) project [6,7], where in addition to island-wide MDA in schools and communities, also behavior change

communication and snail control interventions were implemented [7,8]. In 2017, in many areas of the islands, urogenital schistosomiasis had been eliminated as a public health problem [4,8,9].“(**revised manuscript, lines 120-130**).

3. Zanzibar is constituted by several islands, Unguja and Pemba being the main. However in the present study authors only chose Pemba. Can the authors point out the motives behind the choice of the mention island leaving alone others e.i. Unguja which is large and densely populated

The study was implemented in Pemba (and not in Unguja) first and foremost due to limited funding, which was not sufficient to include both islands, but only 20 administrative areas in the North of Pemba Island. Second, the Swiss Tropical Public Health Institute has a long-standing and very fruitful research partnership with the Public Health Laboratory-Ivo de Carneri in Pemba Island, which was an ideal and well-equipped basis to conduct solid research.

4. Line 230-231: Owing the fact that lack of patients compliance to treatments by praziquantel is among factors that bottlenecked effective control of schistosomiasis in many places. What further initiatives were done to ensure that the advised patients actually get medication? If no, I wonder this could be one of the factors that contributed to the failure to achieve interruption of the disease transmission observed in the present study.

In the low-prevalence implementation units that were the focus of our surveillance-response approach, only positive individuals were treated with praziquantel. In the absence of a highly sensitive and specific point-of-care test for *S. haematobium* diagnosis, for test-and-treat in households and at waterbodies in our study, we used reagent strips to test for microhaematuria as a proxy for infection. Microhaematuria-positive individuals were offered praziquantel directly at the place of testing. Those who tested microhaematuria-negative but were found infected with *S. haematobium* through urine filtration microscopy in the laboratory were followed up for praziquantel treatment. In such cases, individuals were contacted and advised to meet with the study team to receive praziquantel. When this was not feasible, they were instructed to obtain the drugs at the nearest health facility supplied with praziquantel through the study team. As the participating health facilities were part of the study activities, the study team was aware of where praziquantel was available. While we cannot guarantee that all individuals ultimately took the drugs, every effort was made to ensure access to treatment and to inform participants. We have added a statement in the discussion to acknowledge compliance as a potential limitation:

During active surveillance in schools and passive surveillance in health facilities, individuals that were *S. haematobium* and/or microhaematuria-positive received treatment with praziquantel that was administered at the point-of-care and directly observed by the study team or health facility staff, respectively (**revised manuscript, lines 233-235 and 273-276**). During reactive surveillance in households and at waterbodies, participants were either treated directly

at the point-of-care by the study team when microhaematuria-positive, or were treated during an arranged meeting with the study team or referred to a health facility for treatment, when the *S. haematobium* infection was only identified in the laboratory (**revised manuscript, lines 242-246**). In case patients were referred to a health centre, the compliance with treatment was not double-checked. The implications of potential incompliance with treatment on intervention effectiveness is now briefly highlighted in the discussion as follows: "Besides the low to moderate sensitivity of the surveillance-response system to detect and treat cases, also incompliance with praziquantel treatment of infected individuals may have impacted on intervention effectiveness. While infected individuals identified during active and passive surveillance received directly observed treatment at the point-of-care, several infected individuals identified by reactive surveillance were referred to health facilities for treatment. In case these individuals did not seek treatment, they may have contributed to maintaining transmission." (**revised manuscript, lines 693-699**).

Moreover, we added compliance in our conclusion: "Clearly, to achieve complete interruption of *Schistosoma* transmission, in addition to treatment of infected individuals and mollusciciding against intermediate host snails, measures that prevent reinfection such as improvement of the water, sanitation and hygiene (WASH) infrastructure and sustainable behavior change communication to increase compliance with interventions and treatment will be required [1-3]." (**revised manuscript, lines 706-711**).

Line 234: As reported in many studies, the use of chemical snail-control measures my potentially be associated with extended effects to un-intended species. Did the authors consider assessing the impact of the snail control method used to the aquatic ecosystem where study was undertaken? If yes include that information, If not kindly give the reason why?

We agree that the use of chemical molluscicides may potentially have extended effects on non-targeted other species. In our study, however, we did not consider to assess the direct impact of snail control on the aquatic ecosystem. The focus of SchistoBreak was to assess the reduction of urogenital schistosomiasis in humans and not the impact of mollusciciding on the ecosystem.

5. In addition to that, it is very obvious for many African societies to use water from these water bodies for domestic purposes. Did the researchers consider putting mark in the treated water bodies in order to avoid unintended effects to human? If yes indicate in the manuscript.

The leaders of the communities where snail control was conducted were informed by the "snail research team" in the days before niclosamide application in their shehia and asked to extent the information to their community (**see revised manuscript, lines 152-159**). No mark was put in the treated water bodies.

Line 198: The reason behind the choice of the madrassa is well stipulated in the manuscript unlike for the schools. Can the authors indicate clearly the criteria used in the selection of schools i.e. When they say that the largest schools were selected per

implementation unit (IU), what does it mean? Do they refer to population size or Km^2 of the school?

We thank the reviewer for the suggestion to clearly define in the manuscript the indicators based on which the primary schools were chosen. We have now included a definition of "largest school" in the revised manuscript: "In each IU, the largest public primary school was chosen, defined as the school with the highest number of enrolled children among all public primary schools in the IU.“ (**revised manuscript, lines 211-213**).

6. If I understand clearly, the primary objective of the study was to intervene the disease on the basis of the surveillance data. If that the case, can the authors explain why they have extended the application of niclosomide even to the places where snails were not detected, keeping in mind that moluscides have severally repotted to have effects to non targeted species, hence its application should be minimized/limited.

We thank the reviewer for this thoughtful comment and the opportunity to clarify. The WHO recommends the use of the molluscicide niclosamide for schistosomiasis elimination and to apply it in known transmission sites [11]. The aim of our study was to break transmission of schistosomiasis. Hence, we applied niclosamide to known habitat areas of the intermediate host snail *Bulinus* at places where humans have water contact (**see revised manuscript, lines 254-256**). If no *Bulinus* were found at a present or past snail survey, niclosamide was not applied (**see revised manuscript, lines 261-262**).

Line 238-239: In addition to data regarding presence or absence of *Bulinus* snails, other information such as water chemistry, and the presence and type of water vegetation were also collected. However, I don't see any where in the manuscript discussion regarding association of this factors and existence or abundance of *Bulinus* snails.

Indeed, we did collect data on the presence or absence of *Bulinus* snails, water chemistry, and vegetation. However, due to the large amount of data collected, a separate publication [12] was dedicated to snail control interventions, including the detailed analysis of environmental and malacological data (**revised manuscript, reference 21**). This separate publication is highlighted and cited in the manuscript: "Details of snail surveys and control are provided elsewhere [12]." (**revised manuscript, lines 262-263**).

7. Line 249: Can the authors explain how they have measures the outcome of the passive surveillance intervention and include such information in the manuscript? Eg. Does the number of diagnosed individuals increased following intervention? etc.

We thank the reviewer for this important comment. The main objective of the study was to interrupt *S. haematobium* transmission through a combination of interventions, including passive surveillance in health facilities. Passive surveillance was therefore not evaluated as a stand-alone intervention, but rather implemented as an integral component of the overall approach to ensure treatment of as many infected individuals as possible.

8. Line 389: The prevalence (%) for children in primary school at 2022 were not indicate. Kindly, indicate.

We are very grateful to the reviewer for pointing out that we did not indicate the percentage of positive-tested children in the primary schools. We have now added the percentage and the sentence reads: “In 2022, a total of 128/4455 (2.9%) children from primary schools and 25/817 (3.1%) children from madrassas were tested for *S. haematobium* and/or microhematuria in primary schools and identified as positive.” (**revised manuscript, lines 414-417**).

Journal Requirements:

If the reviewer comments include a recommendation to cite specific previously published works, please review and evaluate these publications to determine whether they are relevant and should be cited. There is no requirement to cite these works unless the editor has indicated otherwise.

No recommendations to cite specific previously published works were provided.

1) Please ensure that the CRediT author contributions listed for every co-author are completed accurately and in full.

At this stage, the following Authors/Authors require contributions: Said Mohammed Ali. Please ensure that the full contributions of each author are acknowledged in the "Add/Edit/Remove Authors" section of our submission form.

The list of CRediT author contributions may be found here: https://journals.plos.org/plosntds/s/authorship#loc-author-contributions

We have indicated the full contributions of Said Mohammed Ali in the submission form.

2) We ask that a manuscript source file is provided at Revision. Please upload your manuscript file as a .doc, .docx, .rtf or .tex. If you are providing a .tex file, please upload it under the item type u2018LaTeX Source Fileu2019 and leave your .pdf version as the item type u2018Manuscriptu2019.

We have uploaded the manuscript at Revision as .docx file.

3) Some material included in your submission may be copyrighted. According to PLOSu2019s copyright policy, authors who use figures or other material (e.g., graphics, clipart, maps) from another author or copyright holder must demonstrate or obtain permission to publish this material under the Creative Commons Attribution 4.0 International (CC BY 4.0) License used by PLOS journals. Please closely review the details of PLOSu2019s copyright requirements here: PLOS Licenses and Copyright. If you need to request permissions from a copyright

holder, you may use PLOS's Copyright Content Permission form. Please respond directly to this email and provide any known details concerning your material's license terms and permissions required for reuse, even if you have not yet obtained copyright permissions or are unsure of your material's copyright compatibility. Once you have responded and addressed all other outstanding technical requirements, you may resubmit your manuscript within Editorial Manager.

Please see our responses below.

Potential Copyright Issues:

i) Figure 2. Please (a) provide a direct link to the base layer of the map (i.e., the country or region border shape) and ensure this is also included in the figure legend; and (b) provide a link to the terms of use / license information for the base layer image or shapefile. We cannot publish proprietary or copyrighted maps (e.g. Google Maps, Mapquest) and the terms of use for your map base layer must be compatible with our CC BY 4.0 license.
Note: if you created the map in a software program like R or ArcGIS, please locate and indicate the source of the basemap shapefile onto which data has been plotted.
If your map was obtained from a copyrighted source please amend the figure so that the base map used is from an openly available source. Alternatively, please provide explicit written permission from the copyright holder granting you the right to publish the material under our CC BY 4.0 license.
If you are unsure whether you can use a map or not, please do reach out and we will be able to help you. The following websites are good examples of where you can source open access or public domain maps:
* U.S. Geological Survey (USGS) - All maps are in the public domain. (http://www.usgs.gov)
* PlaniGlobe - All maps are published under a Creative Commons license so please cite u201cPlaniGlobe, http://www.planiglobe.com, CC BY 2.0u201d in the image credit after the caption. (http://www.planiglobe.com/?lang=enl)
* Natural Earth - All maps are public domain. (http://www.naturalearthdata.com/about/terms-of-use/).

The following information is provided in the figure caption of "Figure 2: The image base map (United Republic of Tanzania – Subnational administrative boundaries) was downloaded from the UN Office for the Coordination of Humanitarian Affairs (OCHA) services (https://data.humdata.org/dataset/cod-ab-tza). The data source is: Tanzania National Bureau of Statistics/UN OCHA ROSA. The data are published under the following license: Creative Commons Attribution for Intergovernmental Organizations (CC BY-IGO; (https://creativecommons.org/licenses/by/3.0/igo/legal code)). Additionally, we received written permission to use and adapt the data from OCHA." If this information is not sufficient, please let us know what exactly is missing.

4) We note that your Data Availability Statement is currently as follows: "All relevant data are within the manuscript and its Supporting Information files.". Please confirm at this time whether or not your submission contains all raw data required to replicate the results of your study. Authors must share the “minimal data set” for their submission. PLOS defines the minimal data set to consist of the data required to replicate all study findings reported in the article, as well as related metadata and methods (https://journals.plos.org/plosone/s/data-availability#loc-minimal-data-set-definition).
For example, authors should submit the following data:
1) The values behind the means, standard deviations and other measures reported;
2) The values used to build graphs;
3) The points extracted from images for analysis..
Authors do not need to submit their entire data set if only a portion of the data was used in the reported study.
If your submission does not contain these data, please either upload them as Supporting Information files or deposit them to a stable, public repository and provide us with the relevant URLs, DOIs, or accession numbers. For a list of recommended repositories, please see https://journals.plos.org/plosone/s/recommended-repositories.
If there are ethical or legal restrictions on sharing a de-identified data set, please explain them in detail (e.g., data contain potentially sensitive information, data are owned by a third-party organization, etc.) and who has imposed them (e.g., an ethics committee). Please also provide contact information for a data access committee, ethics committee, or other institutional body to which data requests may be sent. If data are owned by a third party, please indicate how others may request data access.
We confirm that our submission includes a minimal data set consisting of the data required to replicate all study findings reported in the article.

Figure resubmission:

While revising your submission, we strongly recommend that you use PLOS’s NAAS tool (https://ngplosjournals.pagemajik.ai/artanalysis) to test your figure files. NAAS can convert your figure files to the TIFF file type and meet basic requirements (such as print size, resolution), or provide you with a report on issues that do not meet our requirements and that NAAS cannot fix.

After uploading your figures to PLOS’s NAAS tool - https://ngplosjournals.pagemajik.ai/artanalysis, NAAS will process the files provided and display the results in the "Uploaded Files" section of the page as the processing is complete. If the uploaded figures meet our requirements (or NAAS is able to fix the files to meet our requirements), the figure will be marked as "fixed" above. If NAAS is unable to fix the files, a red "failed" label will appear above. When NAAS has confirmed that the figure files meet our requirements, please download the file via the download option, and include these NAAS processed figure files when submitting your revised manuscript.

We used the PLOS's NAAS tool (https://ngplosjournals.pagemajik.ai/artanalysis) to test our figure files as recommended and downloaded the "fixed" files. We submitted these NAAS processed figure files together with our revised manuscript.

Reproducibility:

To enhance the reproducibility of your results, we recommend that authors of applicable studies deposit laboratory protocols in protocols.io, where a protocol can be assigned its own identifier (DOI) such that it can be cited independently in the future. Additionally, PLOS ONE offers an option to publish peer-reviewed clinical study protocols. Read more information on sharing protocols at https://plos.org/protocols?utm_medium=editorial-email&utm_source=authorletters&utm_campaign=protocols

Our study protocol is published, please see: https://bmcinfectdis.biomedcentral.com/articles/10.1186/s12879-021-06620-8.

1. Grimes JET, Croll D, Harrison WE, Utzinger J, Freeman MC, Templeton MR. The roles of water, sanitation and hygiene in reducing schistosomiasis: A review. Parasit Vectors. 2015;8(1): 156.
2. Trippler L, Ali SM, Ali MN, Mohammed UA, Suleiman KR, Ndum NC, et al. Adaptive integrated intervention approaches for schistosomiasis elimination in Pemba: A 4-year intervention study and focus on hotspots. PLoS Negl Trop Dis. 2025;19(5).
3. Lund AJ, Sam MM, Sy AB, Sow OW, Ali S, Sokolow SH, et al. Unavoidable risks: Local perspectives on water contact behavior and implications for schistosomiasis control in an agricultural region of northern Senegal. Am J Trop Med Hyg. 2019;101(4): 837-847.
4. Trippler L, Knopp S, Welsche S, Webster BL, Stothard JR, Blair L, et al. The long road to schistosomiasis elimination in Zanzibar: A systematic review covering 100 years of research, interventions and control milestones. Adv Parasitol. 2023.
5. World Health Organization. Global health estimates 2021: Disease burden by cause, age, sex, by country and by region, 2000-2021. In: editor.^editors. ed. Geneva: 2024. p.
6. Knopp S, Mohammed KA, Ali SM, Khamis IS, Ame SM, Albonico M, et al. Study and implementation of urogenital schistosomiasis elimination in Zanzibar (Unguja and Pemba islands) using an integrated multidisciplinary approach. BMC Public Health. 2012;12: 930.
7. Knopp S, Person B, Ame SM, Ali SM, Hattendorf J, Juma S, et al. Evaluation of integrated interventions layered on mass drug administration for urogenital schistosomiasis elimination: A cluster-randomised trial. The Lancet Global Health. 2019;7(8): e1118-e1129.
8. Knopp S, Ame SM, Person B, Hattendorf J, Rabone M, Juma S, et al. A 5-year intervention study on elimination of urogenital schistosomiasis in Zanzibar:

Parasitological results of annual cross-sectional surveys. PLoS Negl Trop Dis. 2019;13(5).

9. Trippler L, Ame SM, Hattendorf J, Juma S, Abubakar S, Ali SM, et al. Impact of seven years of mass drug administration and recrudescence of *Schistosoma haematobium* infections after one year of treatment gap in Zanzibar: Repeated cross-sectional studies. PLoS Negl Trop Dis. 2021;15(2): e0009127. Epub 2021/02/13.
10. Knopp S, Person B, Ame SM, Ali SM, Muhsin J, Juma S, et al. Praziquantel coverage in schools and communities targeted for the elimination of urogenital schistosomiasis in Zanzibar: A cross-sectional survey. Parasit Vectors. 2016;9(1).
11. World Health Organization. Field use of molluscicides in schistosomiasis control programmes - an operational manual for programme managers. 2017 Report No.: Contract No.
12. Trippler L, Ali SM, Masoud MO, Mohammed ZH, Amour AK, Suleiman KR, et al. Impact of chemical snail control on intermediate host snail populations for urogenital schistosomiasis elimination in Pemba, Tanzania: findings of a 3-year intervention study. Parasit Vectors. 2024;17(1): 489.
