# Supplementary material for: Evaluation of surveillance-response interventions for Schistosoma haematobium elimination on Pemba Island, Tanzania: A 4-year intervention study with repeated cross-sectional surveys
Source: PLoS Negl Trop Dis. 2026 Feb 3;20(2):e0013947. doi: 10.1371/journal.pntd.0013947 (PMC12867264; doi:10.1371/journal.pntd.0013947)
Supplement: Attachment — Submitted filename: PNTD-D-25-00990R1_Response to Reviewers_16.01.2025.pdf [file pntd.0013947.s008.pdf]

Trippler et al. **“**Evaluation of surveillance-response interventions for *Schistosoma haematobium* elimination on Pemba Island, Tanzania: a 4-year intervention study with repeated cross-sectional surveys” (Your reference no. PNTD-D-25-00990R1) – **Point-by-Point response**

Allschwil, 16 January 2026

Dear Dr Tamarozzi,

We were delighted to receive your e-mail dated 17 December 2025, which provided renewed and very valuable feedback from Reviewer#1 regarding the afore mentioned manuscript. Over the past weeks, we carefully revised the manuscript in light of the reviewer’s comments and suggestions and feel that the overall quality of our manuscript has been further improved.

Below, please find our point-by-point response, clearly indicating how and where in the manuscript changes have been made (line numbers referring). To assist you in readily tracking our amendments in the manuscript, we have marked all changes made by using track changes. Additionally, we submit a clean version of the revised manuscript.

We very much hope that this revised version is suitable for publication in *PLoS Neglected Tropical Diseases*. Hence, we look forward to your further disposition.

Yours sincerely,

Lydia Trippler and Stefanie Knopp

(on behalf of all authors)

****

Dear Dr. Knopp,

Thank you for submitting your manuscript to PLOS Neglected Tropical Diseases. After careful consideration, we feel that it has merit but does not fully meet PLOS Neglected Tropical Diseases's publication criteria as it currently stands. Therefore, we invite you to submit a revised version of the manuscript that addresses the points raised during the review process.

We thank you and the two external reviewers very much indeed for the repeated thorough appraisal of our work and the overall positive feedback. We tried our level best to address the remaining points of Reviewer#1 and amended the manuscript accordingly. Reviewer#2 did not provide any additional comments. Please find our point-by-point response below.

Please submit your revised manuscript by Jan 16 2026 11:59PM. If you will need more time than this to complete your revisions, please reply to this message or contact the journal office at plosntds@plos.org. When you're ready to submit your revision, log on to https://www.editorialmanager.com/pntd/ and select the 'Submissions Needing Revision' folder to locate your manuscript file.

We have submitted our revised manuscript along with a point-by-point response to the reviewers' comments on January 16, 2026.

Please include the following items when submitting your revised manuscript:

* A rebuttal letter that responds to each point raised by the editor and reviewer(s). You should upload this letter as a separate file labeled 'Response to Reviewers'. This file does not need to include responses to any formatting updates and technical items listed in the 'Journal Requirements' section below.

We have uploaded a "Response to Reviewers" rebuttal letter, which responds to each point raised by the reviewers to the *PloS Neglected Tropical Diseases* online system.

* A marked-up copy of your manuscript that highlights changes made to the original version. You should upload this as a separate file labeled 'Revised Manuscript with Track Changes'.

We have uploaded a marked-up copy of our manuscript that highlights changes made to the original version using track changes and labelled it "Revised Manuscript with Track Changes".

* An unmarked version of your revised paper without tracked changes. You should upload this as a separate file labeled 'Manuscript'.

We have uploaded an unmarked version of our revised paper without tracked changes and labelled it "Manuscript".

If you would like to make changes to your financial disclosure, competing interests statement, or data availability statement, please make these updates within the submission form at the time of resubmission. Guidelines for resubmitting your figure files are available below the reviewer comments at the end of this letter.

We did not change our financial disclosure, competing interests statement, or data availability statement.

We look forward to receiving your revised manuscript.

Kind regards,

Francesca Tamarozzi
Section Editor
PLOS Neglected Tropical Diseases

Shaden Kamhawi
co-Editor-in-Chief
PLOS Neglected Tropical Diseases
orcid.org/0000-0003-4304-636XX

Paul Brindley
co-Editor-in-Chief
PLOS Neglected Tropical Diseases
orcid.org/0000-0003-1765-0002

****

REVIEWER SUMMARY AND GENERAL COMMENTS

Reviewer #1:

1.The 2021 sensitivity estimate of 114.2% is anomalously high due to data imputation, which undermines the credibility of the three-year overall performance assessment (44.8%). To address this, the manuscript should clearly present the 2022-2023 sensitivity (23.0%) – derived from complete data – as the primary finding in the Results section. Furthermore, the Discussion should explicitly state the uncertainty associated with the 2021 imputed data and critically examine the potential biases introduced by estimating the total number of infections (N_inf) from projected census and survey prevalence data in a low-transmission setting.

We thank Reviewer#1 for once more scrutinizing our manuscript. In line with his/her suggestion, we now provide the sensitivity (23.0%) of the surveillance-response approach from the two periods (2022 and 2023), where complete data were available, as the primary finding in the Results section of the Abstract (**see revised manuscript, lines 47-49**). In the Results section of the main body of the manuscript, we slightly modified Fig 3 and used a hatched column for the bar that indicates the "observed" *S. haematobium* prevalence in 2021, which was derived by a combination of diagnostic assessments and data imputation. Moreover, we clearly indicate that for the 1st period (2021) results were derived from a subgroup of individuals only and that the missing data were imputed (**see revised manuscript, lines 482-487 and lines 497-500**). In addition, we indicate that "The sensitivity of the surveillance interventions to detect *S. haematobium*-infected school-aged children was 234.4% in 2021 (where data were partially imputed), 22.3% in 2022, and 53.5% in 2023. The sensitivity of the surveillance interventions to detect *S. haematobium*-infected adults was 4.2% in 2021, 2.0% in 2022, and 2.8% in 2023." (**see revised manuscript, lines 503 - 507**).

In the Discussion, we now clearly state that "Due to the uncertainty associated with the 2021 imputed data, we consider the overall sensitivity of 23.0% from 2022 and 2023 as accurate" (**see revised manuscript, lines 606-607**).

With regard to the Reviewer's suggestion "to critically examine the potential biases introduced by estimating the total number of infections (N_inf) from projected census and survey prevalence data in a low-transmission setting" we would like to offer the following considerations:

Regarding (N_inf): Besides random error (i.e. sampling variability associated with low prevalences) we might be confronted with two types of systematic error: selection and misclassification bias.

Given that misclassification is likely to be similar in both the cross-sectional survey data and the observed surveillance-response data, in our opinion, misclassification does not warrant further consideration. With regard to selection bias, we revised the discussion as follows: "Fourth, a selection bias might have occurred if the schools and madrassas included in the study were not representative of the target population, e.g. in case many non-school-attending children lived in the area. Moreover, a selection bias might have occurred if *S. haematobium*-infected individuals did comply less with study procedures than non-infected participants, e.g., by providing consent for participation or a urine sample for examination." (**see revised manuscript, lines 617-621**).

We believe that other sources of uncertainty, e.g. assuming a population growth rate of 2.5%, are unlikely to have a substantial impact and can hence be neglected in terms of increasing fluctuation.

2. The study's conclusions are drawn from a specific context in Pemba Island, and their applicability to other low-prevalence regions requires more thorough discussion. To strengthen the paper, a dedicated subsection should be added to the Discussion that outlines the contextual factors of the study setting and thoughtfully discusses the potential adjustments needed for implementing this surveillance-response approach in areas with different health system capacities, resource levels, or environmental conditions.

We thank Reviewer#1 for this suggestion and amended the following subsection: "Our study area in Pemba consisted of 20 neighboring IUs and covered an area of approximately 150 square kilometers. Moreover, the IUs were connected by a good road system, and schools, villages, water bodies and health facilities were mostly easily accessible by car and reachable within two hours from PHL-IdC. To work on a larger scale, across Pemba or in other countries and areas with different health system capacities, resource levels, or environmental conditions, with good coverage and improved sensitivity, elimination programs will need a large number of well-trained staff and adequate tools for surveillance and response activities, data management, and communication." (**see revised manuscript, lines 665-672**).

3.A significant limitation is the lack of data on final treatment completion for laboratory-confirmed cases who were referred to health facilities, which weakens the analysis of why transmission interruption was not achieved. This gap should be explicitly acknowledged in the Limitations section of the Discussion. The authors should discuss how this potential break in the treatment cascade could have influenced the outcome and recommend that future programmatic implementations establish mechanisms to verify treatment adherence for all diagnosed cases.

We thank Reviewer#1 for this suggestion and amended the discussion as follows: "While infected individuals identified during active and passive surveillance received directly observed treatment at the point-of-care, several infected individuals identified by reactive surveillance were referred to health facilities for treatment. In case these infected individuals did not seek treatment, they may have contributed to maintaining transmission. Such an interruption in the treatment cascade may hence have influenced the outcome in our study and future programmatic implementations should establish robust mechanisms to ensure and verify treatment adherence for all diagnosed cases." (**see revised manuscript, lines 695-702**).

Figure resubmission:

While revising your submission, we strongly recommend that you use PLOS's NAAS tool (https://ngplosjournals.pagemajik.ai/artanalysis) to test your figure files. NAAS can convert your figure files to the TIFF file type and meet basic requirements (such as print size, resolution), or provide you with a report on issues that do not meet our requirements and that NAAS cannot fix.

After uploading your figures to PLOS's NAAS tool - https://ngplosjournals.pagemajik.ai/artanalysis, NAAS will process the files provided and display the results in the "Uploaded Files" section of the page as the processing is complete. If the uploaded figures meet our requirements (or NAAS is able to fix the files to meet our requirements), the figure will be marked as "fixed" above. If NAAS is unable to fix the files, a red "failed" label will appear above. When NAAS has confirmed that the figure files meet our requirements, please download the file via the download option, and include these NAAS processed figure files when submitting your revised manuscript.

We used the PLOS's NAAS tool (https://ngplosjournals.pagemajik.ai/artanalysis) to test our figure files as recommended and downloaded the "fixed" files. We submitted these NAAS processed figure files together with our revised manuscript.

Reproducibility:

To enhance the reproducibility of your results, we recommend that authors of applicable studies deposit laboratory protocols in protocols.io, where a protocol can be assigned its own identifier (DOI) such that it can be cited independently in the future. Additionally, PLOS ONE offers an

option to publish peer-reviewed clinical study protocols. Read more information on sharing protocols at https://plos.org/protocols?utm_medium=editorial-email&utm_source=authorletters&utm_campaign=protocols

Our study protocol is published in *BMC Infectious Diseases*, please see: https://bmcinfectdis.biomedcentral.com/articles/10.1186/s12879-021-06620-8.
