# Supplementary material for: Evaluation of surveillance-response interventions for Schistosoma haematobium elimination on Pemba Island, Tanzania: A 4-year intervention study with repeated cross-sectional surveys
Source: PLoS Negl Trop Dis. 2026 Feb 3;20(2):e0013947. doi: 10.1371/journal.pntd.0013947 (PMC12867264; doi:10.1371/journal.pntd.0013947)
Supplement: Attachment — Submitted filename: Comments plos.docx [file pntd.0013947.s006.docx]

Comments

1. Line 87: Authors have pointed out that sub-saharan African is the highly affected region. In view of this, it will be worthy including recent data that portrayed the magnitude of the disease in the mentioned region and its contribution to the global schistosomiasis prevalence.
2. The introduction section is very brief; I therefore suggest the inclusion of a brief history of schistosomiasis control in Zanzibar which consequently led to the diseases elimination.
3. Zanzibar is constituted by several islands, Unguja and Pemba being the main. However in the present study authors only chose Pemba. Can the authors point out the motives behind the choice of the mention island leaving alone others e.i. Unguja which is large and densely populated
4. Line 230-231: Owing the fact that lack of patients compliance to treatments by praziquantel is among factors that bottlenecked effective control of schistosomiasis in many places. What further initiatives were done to ensure that the advised patients actually get medication? If no, I wonder this could be one of the factors that contributed to the failure to achieve interruption of the disease transmission observed in the present study.
5. Line 234: As reported in many studies, the use of chemical snail-control measures my potentially be associated with extended effects to un-intended species. Did the authors consider assessing the impact of the snail control method used to the aquatic ecosystem where study was undertaken? If yes include that information, If not kindly give the reason why?
6. In addition to that, it is very obvious for many African societies to use water from these water bodies for domestic purposes. Did the researchers consider putting mark in the treated water bodies in order to avoid unintended effects to human? If yes indicate in the manuscript.
7. Line 198: The reason behind the choice of the madrassa is well stipulated in the manuscript unlike for the schools. Can the authors indicate clearly the criteria used in the selection of schools i.e. When they say that the largest schools were selected per implementation unit (IU), what does it mean? Do they refer to population size or Km^2^ of the school?
8. If I understand clearly, the primary objective of the study was to intervene the disease on the basis of the surveillance data. If that the case, can the authors explain why they have extended the application of niclosomide even to the places where snails were not detected, keeping in mind that moluscides have severally repotted to have effects to non targeted species, hence its application should be minimized/limited.
9. Line 238-239: In addition to data regarding presence or absence of *Bulinus* snails, other information such as water chemistry, and the presence and type of water vegetation were also collected. However, I don’t see any where in the manuscript discussion regarding association of this factors and existence or abundance of *Bulinus* snails.
10. Line 249: Can the authors explain how they have measures the outcome of the passive surveillance intervention and include such information in the manuscript? Eg. Does the number of diagnosed individuals increased following intervention? etc.
11. Line 389: The prevalence (%) for children in primary school at 2022 were not indicate. Kindly, indicate.
